# One-year Longitudinal Study of Antibody Profiles in Children: MIS-C Versus Children With Uncomplicated COVID-19

**DOI:** 10.1097/INF.0000000000004261

**Published:** 2024-02-02

**Authors:** Meriem Bekliz, Anaïs Thiriard, Silvia Stringhini, Arnaud Marchant, Arnaud M. Didierlaurent, Benjamin Meyer, Geraldine Blanchard-Rohner

**Affiliations:** Department of Microbiology and Molecular Medicine, University of Geneva, Geneva, Switzerland; European Plotkin Institute for Vaccinology, Université libre de Bruxelles, Brussels, Belgium; Population Epidemiology Unit, Primary Care Division, University of Geneva, Geneva, Switzerland; European Plotkin Institute for Vaccinology, Université libre de Bruxelles, Brussels, Belgium; Centre of Vaccinology, Department of Pathology and Immunology, University of Geneva, Geneva, Switzerland; Centre of Vaccinology, Department of Pathology and Immunology, University of Geneva, Geneva, Switzerland, Unit of Pediatric Immunology, Rheumatology and Vaccinology, Children’s Hospital of Geneva, Geneva University Hospitals and Faculty of Medicine, Geneva, Switzerland

## To the Editors:

In the spring of 2020, most children presented with an asymptomatic or pauci-symptomatic infection to SARS-CoV-2,^1^ while a subset of children developed a multisystem inflammatory syndrome (MIS-C). The physiopathology of MIS-C has been widely studied, but the exact cause leading to MIS-C development in some children is still unclear. We have shown previously that 1-month postinfection, the neutralizing activity of circulating antibodies and the level of serum IgA against SARS-CoV-2 were higher in MIS-C patients compared with children with uncomplicated COVID-19.^2^ In contrast, no difference was observed in the levels of IgG and IgM antibodies. Here, we assessed whether the specific features of the antibody response of MIS-C children were sustained throughout a year after infection.

The MIS-C children had blood sampling at 1-month (first day of hospitalization), 6 months and 12 months postinfection (CCER 2020-00835). Control children with uncomplicated COVID-19 were drawn from the “understanding COVID” study (CCER 2020-00516)^3^ for the baseline (T0), T6 and T12 examinations and from the SEROCOV-KIDS (CCER 2021-01973) for the T12 examination.^4^ Importantly, both the MIS-C children and the controls had been infected during the same time-period, when the original strain and the antigenically similar alpha variant of SARS-CoV-2 circulated in Switzerland. Children who had been vaccinated during the study were excluded from the analysis. Methodological details were previously described.^2^

**FIGURE 1.**
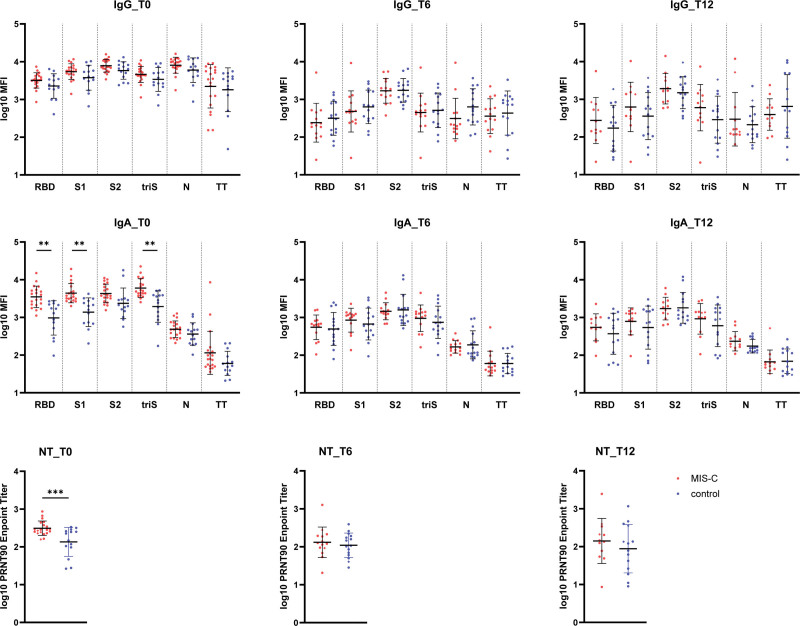
Antibody response against SARS-CoV-2 in MIS-C and control children. IgG (upper line) and IgA (middle line) antibody levels against SARS-CoV-2 receptor binding domain (RBD), S1 and S2 domain of the spike protein, trimerized full-length spike (triS) and nucleocapsid protein (N) as well as a tetanus toxoid (TT) in MIS-C (red dots) and control children (blue dots) at 1-month postinfection and then at 6 months and 12 months. SARS-CoV-2 specific neutralizing antibodies determined by plaque reduction neutralization assay in MIS-C and control children (lower line).

IgA antibodies levels underwent a rapid decrease at 6 months postinfection and remained stable at 12 months. There was no apparent difference in IgA antibodies levels and trajectories between the MIS-C children and children with uncomplicated COVID-19. At 6 months, MIS-C children reached neutralization titers like those of children with uncomplicated COVID-19 and the levels remained stable by 12 months postinfection. IgG antibody levels decreased slowly for most of the SARS-CoV-2 antigens at 6 months and 12 months in both groups. The level of S2-specific antibodies, however, remained higher than those of other IgGs (see Fig. 1).

Overall, IgG, IgA and neutralizing antibodies against SARS-CoV-2 remain high up to 1-year postinfection, irrespective of whether children had developed MIS-C. The fact that the initially strongest IgA and neutralizing antibody response was lost at 6 months postinfection, suggests that those previous observations probably reflect the consequences of the gastrointestinal inflammation, which is a prominent feature of MIS-C children.^5^

Further studies are still required to elucidate the pathophysiology of MIS-C and the factors predisposing individuals to develop MIS-C. We show that humoral immunity is maintained similarly in MIS-C and uncomplicated COVID-19 children, suggesting that both groups remain protected from an episode of MIS-C since fewer cases were reported upon immunization in children. In addition, almost no MIS-C cases were reported with the last circulating strains.

## References

[1] Cui X Zhao Z Zhang T. A systematic review and meta-analysis of children with coronavirus disease 2019 (COVID-19). J Med Virol. 2021;93:1057–1069. 32761898 10.1002/jmv.26398 PMC7436402

[2] Thiriard A Meyer B Eberhardt CS. Antibody response in children with multisystem inflammatory syndrome related to COVID-19 (MIS-C) compared to children with uncomplicated COVID-19. Front Immunol. 2023;14:1107156. 37006315 10.3389/fimmu.2023.1107156 PMC10050384

[3] Vono M Huttner A Lemeille S. Robust innate responses to SARS-CoV-2 in children resolve faster than in adults without compromising adaptive immunity. Cell Rep. 2021;37:109773. 34587479 10.1016/j.celrep.2021.109773 PMC8440231

[4] Stringhini S Zaballa ME Perez-Saez J; Specchio-COVID19 Study Group. Seroprevalence of anti-SARS-CoV-2 antibodies after the second pandemic peak. Lancet Infect Dis. 2021;21:600–601. 33539733 10.1016/S1473-3099(21)00054-2 PMC8063076

[5] Feldstein LR Rose EB Horwitz SM; Overcoming COVID-19 Investigators. Multisys-tem inflammatory syndrome in US children and adolescents. N Engl J Med. 2020;383:334–346. 32598831 10.1056/NEJMoa2021680 PMC7346765

